# Chemometric Discrimination of the Geographical Origin of *Rheum tanguticum* by Stable Isotope Analysis

**DOI:** 10.3390/foods13193176

**Published:** 2024-10-06

**Authors:** Bayan Nuralykyzy, Jing Nie, Guoying Zhou, Hanyi Mei, Shuo Zhao, Chunlin Li, Karyne M. Rogers, Yongzhi Zhang, Yuwei Yuan

**Affiliations:** 1 State Key Laboratory for Managing Biotic and Chemical Threats to the Quality and Safety of Agro-Products, Zhejiang Academy of Agricultural Sciences, Hangzhou 310021, China; baiana2020@nwafu.edu.cn (B.N.);; 2 Institute of Agro-Products Safety and Nutrition, Zhejiang Academy of Agricultural Sciences, Key Laboratory of Information Traceability for Agricultural Products, Ministry of Agriculture and Rural Affairs of China, Hangzhou 310021, China; 3 Northwest Institute of Plateau Biology, Chinese Academy of Sciences, Xining 810008, China; 4 National Isotope Centre, GNS Science, Lower Hutt 5040, New Zealand

**Keywords:** rhubarb, chemometrics, geographical origin, stable isotopes, isotope ratio mass spectrometry

## Abstract

*Rheum tanguticum* is one of the primary rhubarb species used for food and medicinal purposes, and it has recently been gaining more attention and recognition. This research represents the first attempt to use stable isotopes and elemental analysis via IRMS to identify the geographical origin of *Rheum tanguticum*. A grand total of 190 rhubarb samples were gathered from 38 locations spread throughout the provinces of Gansu, Sichuan, and Qinghai in China. The carbon content showed a decreasing trend in the order of Qinghai, followed by Sichuan, and then Gansu. Nitrogen content was notably higher, with Qinghai and Sichuan displaying similar levels, while Gansu had the lowest nitrogen levels. Significant differences were noted in the *δ*^13^C (−28.9 to −26.5‰), *δ*^15^N (2.6 to 5.6‰), *δ*^2^H (−120.0 to −89.3‰), and *δ*^18^O (16.0‰ to 18.8‰) isotopes among the various rhubarb cultivation areas. A significant negative correlation was found between %C and both longitude and humidity. Additionally, *δ*^13^C and *δ*^15^N isotopes were negatively correlated with longitude, and *δ*^15^N showed a negative correlation with humidity as well. *δ*^2^H and *δ*^18^O isotopes exhibited a strong positive correlation with latitude, while significant negative correlations were observed between *δ*^2^H and *δ*^18^O isotopes and temperature, precipitation, and humidity. The LDA, PLS-DA, and k-NN models all exhibited strong classification performance in both the training and validation sets, achieving accuracy rates between 82.1% and 91.7%. The combination of stable isotopes, elemental analysis, and chemometrics provides a reliable and efficient discriminant model for accurately determining the geographical origin of *R. tanguticum* in different regions. In the future, the approach will aid in identifying the geographical origin and efficacy of rhubarb in other studies.

## 1. Introduction

*Rheum tanguticum* (*R. tanguticum*) is a widely consumed natural plant, both as food and in medicine, native to northwestern and southwestern China, and primarily distributed across the provinces of Gansu, Qinghai, and Sichuan [1,2]. As a food, rhubarb is commonly used in pies and desserts for its fruity flavor, although in some countries it is eaten as a vegetable. Rhubarb provides health benefits and is also utilized in the production of alcoholic beverages [3]. The medicinal use of rhubarb stalks dates back roughly 5000 years. In traditional Chinese medicine (TMC), rhubarb is known as the “general” because it is used in emergency treatments and plays a vital role [4]. Numerous studies have examined the medicinal uses of rhubarb [5,6] with the roots and rhizomes of *R. tanguticum* being recognized for their antibacterial properties [7], as well as their laxative and anti-inflammatory effects [8].

Research has shown that the quality of *R. tanguticum* varies based on its geographical origin [9,10], which may be influenced by environmental factors such as altitude [11], climate [12], and others. Moreover, rhubarb from different regions can vary in quality, and this variability is linked to the methods used for identifying *R. tanguticum*. As a result, it is essential to distinguish rhubarb from different regions and confirm its authenticity. Gansu, Qinghai, and Sichuan are the primary production areas for *R. tanguticum*, and rhubarb from these regions is regarded as being of superior quality. Historically, geographical origin has been used as an indicator of the quality of local herbs, reflecting the holistic approach of traditional Chinese medicine. Understanding the localization of medicinal plants is an important topic in TCM, particularly as the global interest in Chinese traditional medicine grows. Determining the geographical origin of medicinal plants is essential for maintaining the quality and effectiveness of these medicines.

Modern technologies, such as liquid chromatography [13], infrared spectroscopy [14], and fluorescence spectroscopy [15], have been applied to determine the origin of rhubarb. As previously noted, the quality of *R. tanguticum* is affected by environmental conditions, climate, and geographical location. These variables can be effectively traced using stable isotope and elemental (%C and %N) analysis to determine the plant’s origin [16]. Previous studies have employed either stable isotopes [1,17,18] or elemental analysis [19,20] alone to determine the origin of medicinal plants. A combined analysis of stable isotopes and elemental content can enhance the accuracy of regional identification, improving the discriminant model’s precision and stability [13,21,22,23]. Isotope ratio mass spectrometry (IRMS) is the most widely used technique for measuring stable isotope ratios and is a highly effective method for tracing the origins of food and medicinal products [24]. Although stable isotope studies are widespread in agricultural research, their application to medicinal plants remains rare. To date, no studies have combined stable isotope analysis with elemental analysis for the identification of rhubarb. This contribution is the first to explore the quality of *R. tanguticum* using IRMS in combination with light element analysis (i.e., C and N). Given the increasing recognition of rhubarb, it is important to thoroughly study the origins of *R. tanguticum* in its production regions.

This study aims to determine the isotope compositions (*δ*^13^C, *δ*^15^N, *δ*^2^H, and *δ*^18^O) and abundances of light elements (%C and %N) in *R. tanguticum* samples from Gansu, Sichuan, and Qinghai using IRMS with the goal of identifying the reasons for their differences or similarities. Furthermore, a correlation analysis between rhubarb stable isotope compositions and environmental factors was performed to examine how climatic conditions influence the stable isotopes and quality variation in rhubarb. Additionally, chemometric models, such as LDA, k-NN, and PLS-DA, which showed high predictive accuracy for distinguishing *R. tanguticum* from different regions, were developed based on IRMS data. This study offers a reliable method for determining the geographical origin of rhubarb, and its results can be used to inform the planning of *R. tanguticum* cultivation.

## 2. Materials and Methods

### 2.1. Sample Collection and Preparation

As previously noted, the research focuses on three major *R. tanguticum* production regions in China: Gansu, Sichuan, and Qinghai. Gansu is situated in the upper reaches of the Yellow River in northwest China, bordered by Sichuan to the south and Qinghai to the west. The altitude in Gansu averages 1930.96 m above sea level, and the region experiences a mid-latitude steppe climate. Sichuan, with its varied topography, is influenced by the Pacific Ocean monsoon in the southeast and the Indian Ocean monsoon in the southwest. Weather conditions in Sichuan differ significantly, with the eastern part having a humid climate, while the western and mountainous areas are characterized by a drier climate. The region’s average altitude is 1055.81 m above sea level. Qinghai, on the other hand, is known for its low temperatures, dry and windy climate, and oxygen deficiency, all of which contribute to its unique climate. The province is marked by high altitudes, expansive mountainous areas, deserts, and thin air, with an average altitude of 4120.55 m above sea level, storing millions of tons of resources.

**Figure 1:**
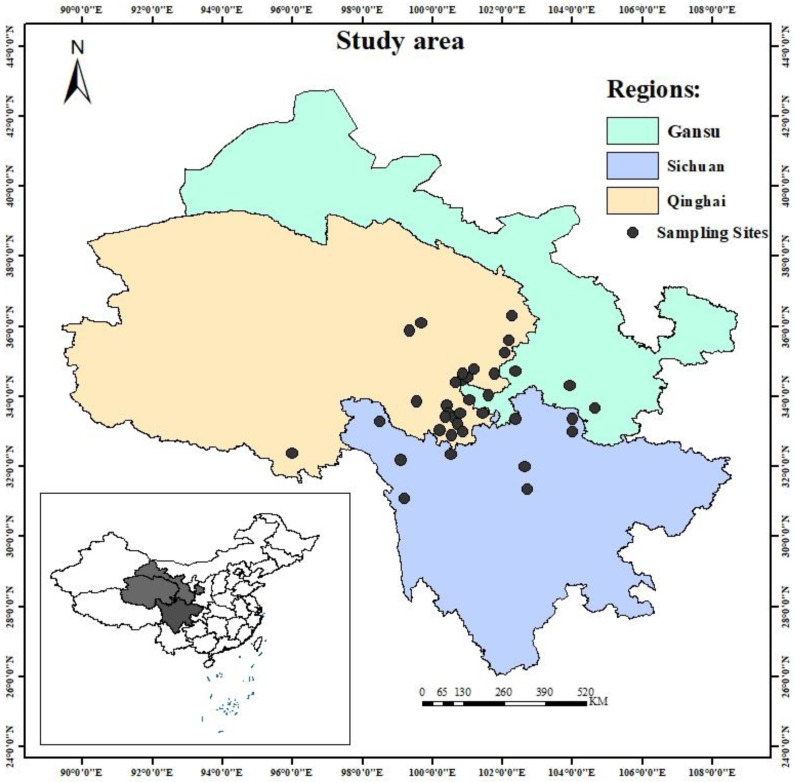
Geographical location of rhubarb samples from different geographical regions in China.

Samples of *R. tanguticum* were collected from Gansu (4 sites), Sichuan (9 sites), and Qinghai (25 sites) between August and September 2018 (Figure 1). In total, samples were collected from 38 sites across the three regions, with five samples taken from each site. This yielded 20 samples from Gansu, 45 from Sichuan, and 125 from Qinghai, for a total of 190 samples (Table S1). The *R. tanguticum* samples were dried in an oven at 40 °C for 4–6 h. After drying, the samples were ground into powder using a ball mill (SCIENTE-48, China) and passed through a 60-mesh sieve. All the dried samples were stored in a desiccator for further analysis.

### 2.2. Stable Isotope Analysis

For the determination of carbon and nitrogen isotopes, approximately 10 mg of each sample was enclosed in a tin cup and combusted in a Vario isotope Cube (Elementar, Langenselbold, Germany) at 1150 °C. The carbon and nitrogen elements were reduced to CO_2_ and N_2_ at 850 °C and then transported by helium gas to a Bio-vision stable isotope ratio mass spectrometer (IRMS) (Elementar, Langenselbold, Germany) for isotope analysis.

For the determination of hydrogen and oxygen isotopes, around 0.5 mg of each sample was placed in a silver cup and pyrolyzed using a Pyro Cube (Elementar, Langenselbold, Germany) at 1450 °C. High-purity hydrogen and oxygen gasses were then obtained and analyzed with an Isoprime 100 isotope ratio mass spectrometer (IRMS) (Elementar, Langenselbold, Germany). The isotope values were calculated using the following equation:

*δ*E = (R_sample_/R_standard_ − 1) (1)

where *δ*E represents *δ*^13^C, *δ*^15^N, *δ*^2^H, and *δ*^18^O, and R_sample_ and R_standard_ are the ratios of ^13^C/^12^C, ^15^N/^14^N, ^2^H/^1^H, or ^18^O/^16^O in the samples and standards, respectively.

Multipoint standards were utilized to calibrate the stable isotope values. The reference standard materials included B2155 (protein, *δ*^13^C = −27‰; *δ*^15^N = 5.9‰) and BCR-657 (*δ*^13^C = −10.8‰) for C and N isotope calibration. The rhubarb samples were also calibrated for C and N isotopes using USGS 64 (glycine, *δ*^13^C = −40.8‰; *δ*^15^N = 1.8‰), USGS-40 (*δ*^13^C = −26.4‰; *δ*^15^N = −4.5‰), and IAEA-N-2 (ammonium Sulfate, *δ*^15^N = 20.3‰). For H and O isotopic calibration, USGS 54 (*δ*^2^H = −150.4‰; *δ*^18^O = 17.8‰) and USGS 56 (*δ*^2^H = −44‰; *δ*^18^O = 27.2‰) standards were used. The accuracy of the analysis, based on the internal quality control of the rhubarb samples, showed a precision of ≤±0.1‰ for *δ*^13^C, ±0.1‰ for *δ*^15^N, ±3‰ for *δ*^2^H, and ±0.3‰ for *δ*^18^O.

### 2.3. Statistical Analysis

Various data analysis methods were employed on the dataset, including one-way ANOVA, linear discriminant analysis (LDA), partial least squares regression discriminant analysis (PLS-DA), and k-nearest neighbor (k-NN). The one-way ANOVA and k-nearest neighbor (k-NN) analysis were conducted using the SPSS software (IBM SPSS Statistics 25), while the partial least squares discriminant analysis (PLS-DA) and linear discriminant analysis (LDA) were performed using the XLSTAT program. The Pearson correlation analysis between stable isotopes and environmental factors was carried out using the Origin software. One-way ANOVA, coupled with Duncan’s test, was used to assess the statistical significance between the rhubarb samples from the different growing regions. Box plots, in combination with Duncan’s test, were employed to evaluate isotopic variations across these regions. LDA, k-NN, and PLS-DA models were applied to classify the geographical origin of the rhubarb samples, and segmented cross-validation was used on all the samples to validate the performance of these models.

## 3. Results and Discussion

### 3.1. Stable Isotope Variations in Rhubarb Samples from Different Regions

**Figure 2:**
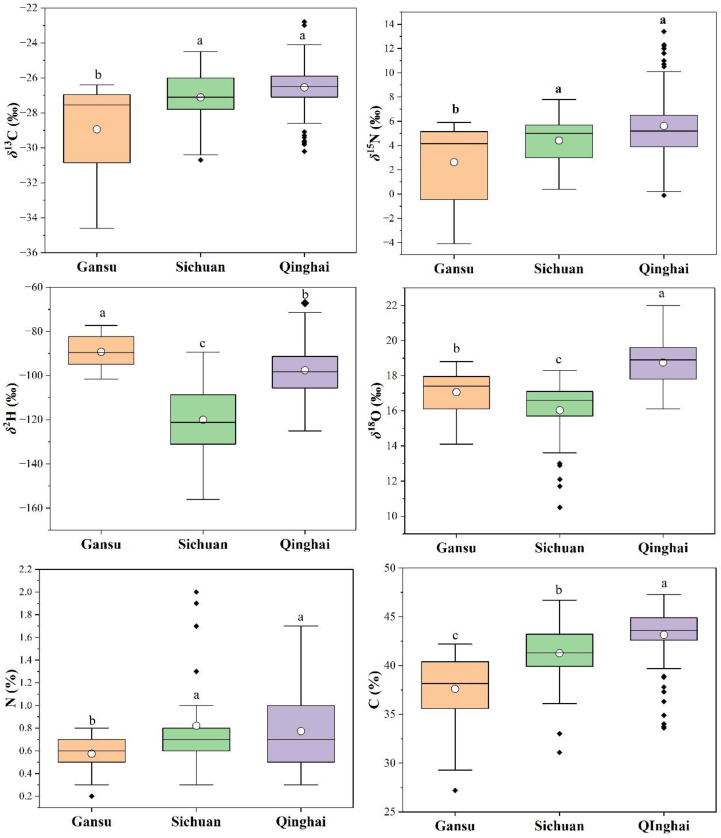
Box and whisker diagram of carbon (*δ*^13^C), nitrogen (*δ*^15^N), hydrogen (*δ*^2^H), and oxygen (*δ*^18^O) isotope compositions and nitrogen (N) and carbon (C) elemental contents of rhubarb in the different geographical regions. The box represents the 25 to 75 percentage, and the center line is the median line. The whiskers represent the range, the black rhombus “♦” is the outlier value, and the white circles “○” represent the mean value. a–c letters represent significant differences.

Stable isotopes such as C, N, H, and O are frequently utilized to trace the geographical origin of plants. Carbon isotopes are linked to plant metabolism during photosynthesis, while isotopes in plants depend on factors like chemical fertilizers, climatic conditions, and soil compositions. H and O isotope ratios reflect the characteristics of water sources absorbed by plants [18,25]. In this study, the *δ*^13^C, *δ*^15^N, *δ*^2^H, *δ*^18^O, %C, and %N contents of the rhubarb samples from the Gansu, Sichuan, and Qinghai regions were determined and are displayed in Figure 2.

Slightly higher *δ*^13^C values were observed in the rhubarb samples from Qinghai (−26.5‰) and Sichuan (−27.1‰), while lower *δ*^13^C values were found in Gansu province (−28.9‰). The *δ*^15^N values ranged from 2.6 to 5.6‰, with higher values in the Qinghai and Sichuan samples, and lower values in Gansu. The variation in *δ*^13^C and *δ*^15^N values may be attributed to Gansu’s northern location, where cooler air temperature could influence isotopic composition. The Sichuan region, influenced by the warm, temperate climate of the Sichuan Basin, shows less negative *δ*^13^C values. In contrast, Qinghai’s arid and semi-arid climates significantly influence the *δ*^13^C values of local vegetation. Due to water stress, plants in these regions tend to close their stomata to reduce water loss, leading to less negative *δ*^13^C values. Other factors such as humidity, longitude, and latitude [26] also influence carbon isotopic composition. The high-altitude Qinghai–Tibet Plateau in Qinghai experiences lower temperatures, which can lead to more negative *δ*^13^C values in local vegetation due to changes in photosynthetic rates.

Qinghai and Sichuan exhibited the highest N isotope composition values, influenced by N sources, soil processes, fertilizer use, and anthropogenic activities. The *δ*^15^N values are affected by soil processes like nitrogen mineralization, nitrification, and denitrification, which depend on temperature, humidity, and altitude. The high-altitude Qinghai–Tibetan plateau tends to enrich *δ*^15^N values in plant tissues due to limited N availability and more intensive N cycling [27]. In the Sichuan Basin, intensive agriculture is often associated with the use of synthetic fertilizers, which can alter *δ*^1^^5^N values in soil and plants. The use of manure or organic fertilizers typically results in higher *δ*^1^^5^N values. Therefore, the differences between the three regions are likely a result of varying climatic conditions, which impact both plants and soil, thereby influencing carbon and nitrogen storage.

The *δ*^2^H values of the rhubarb varied across the regions, with the highest average value observed in Gansu (−89.3‰), followed by Qinghai (−97.6‰), and the lowest in Sichuan (−120.0‰). The higher *δ*^2^H values in Gansu can be attributed to its northern location, where colder and drier conditions prevail. Previous studies [28,29,30] have indicated that *δ*^2^H values tend to decrease with increasing distance from the coast. As Qinghai and Sichuan are further inland compared to Gansu, this geographical factor likely contributes to their lower *δ*^2^H values. The *δ*^18^O values showed slight variation between the regions, with the following decreasing trend in average values: Qinghai > Gansu > Sichuan (Figure 2).

The isotopic composition of hydrogen and oxygen is generally influenced by climatic and environmental factors [31], such as temperature [26], precipitation [32], and humidity [33]. Based on the climatic data from August to September, Qinghai experienced lower temperatures, precipitation, and humidity compared to Gansu and Sichuan, which may account for the lower *δ*^18^O and *δ*^2^H values in Qinghai. In colder environments, more negative isotope values are observed [28], as lower temperatures lead to the preferential removal of the hydrogen isotope condensation, resulting in a more negative *δ*^2^H value. This is particularly evident in the Qinghai–Tibetan Plateau, where colder winter temperatures cause *δ*^2^H depletion [34], leading to more negative *δ*^2^H values in precipitation.

The Gansu region receives relatively little annual precipitation, leading to less *δ*^2^H depletion, and therefore, less negative isotope values. Conversely, Sichuan receives more precipitation, resulting in a stronger “quantity effect”, producing more negative *δ*^2^H values in precipitation. Humidity also plays a key role in the isotopic composition of *δ*^2^H and *δ*^1^^8^O in precipitation. In low humidity conditions, higher evaporation leads to more negative isotope values. The high humidity in the Sichuan Basin limits evaporation, contributing to more negative *δ*^2^H values in precipitation, whereas the low humidity in Qinghai leads to the evaporative enrichment of *δ*^2^H and *δ*^1^^8^O, resulting in higher isotope values in Qinghai precipitation.

The mean C content of the rhubarb differed significantly between the regions, with the highest value observed in Qinghai (43.2%), followed by Sichuan (41.3%), and the lowest in Gansu (37.6%). N content was slightly higher and comparable in the Qinghai and Sichuan regions (0.8%), while Gansu had the lowest value (0.6%). These findings suggest that the elemental and stable isotope compositions of *R. tanguticum* vary geographically across the production regions, likely due to climatic variations that influence the rhubarb quality in Gansu, Qinghai, and Sichuan.

### 3.2. Correlation Analysis of Stable Isotopes with Environmental Factors

**Figure 3:**
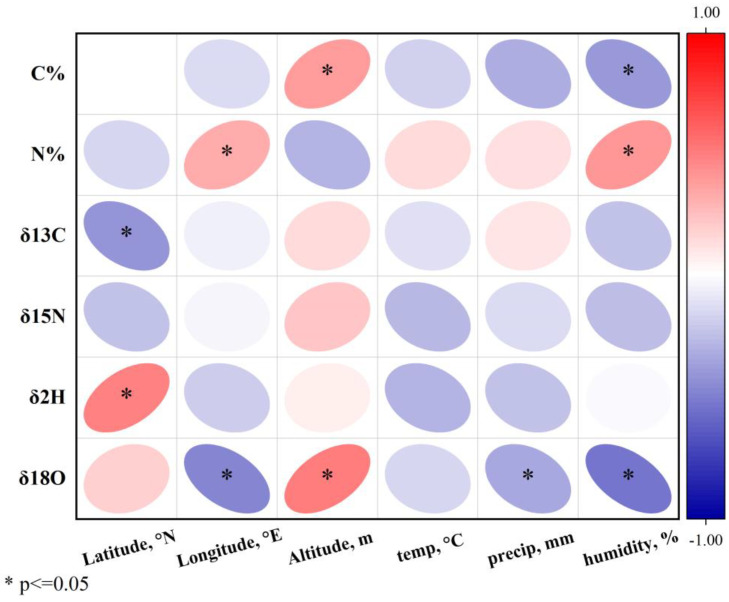
Correlation plots of stable isotope values and environmental factors.

As reported in previous studies, the quality and effects of rhubarb, including *R. tanguticum,* are influenced by environmental factors [12,13], such as temperature, humidity, precipitation, as well as latitude, longitude, altitude [14,35], and seasonal variations [36,37]. In this study, a correlation analysis was performed between the indicators (C, N, *δ*^13^C, *δ*^15^N, *δ*^2^H, and *δ*^18^O) of *R. tanguticum* and environmental factors such as latitude, longitude, altitude, temperature, precipitation, and humidity to evaluate the influence of climatic conditions on stable isotope compositions. The correlation results are illustrated in Figure 3, while the climatic conditions during the sampling period across the three production regions are outlined in Table S2 of the Supplementary Materials.

The significant negative correlation between the carbon content and both longitude and humidity (*p* < 0.05) indicates that these environmental factors exert the greatest influence on the carbon content. This relationship indicates a consistent decrease in the carbon content with increasing longitude, which may be linked to regional variations or environmental stressors along longitudinal gradients. The negative correlation with humidity suggests that higher humidity levels may reduce the carbohydrate content, possibly due to effects on photosynthesis or plant metabolism. Similar findings from previous studies have shown that humidity and geographic location can influence carbohydrate accumulation in plants by affecting physiological processes such as water availability and carbon assimilation [38,39].

The negative correlation between *δ*^13^C and *δ*^15^N isotopes and longitude (*p* < 0.05) highlights the spatial variability of stable isotopic compositions across geographic regions. *δ*^13^C reflects photosynthetic pathways and water use efficiency, which may vary along longitudinal gradients due to changing climate and environmental conditions. The negative correlation between *δ*^15^N and longitude suggests potential changes in nitrogen cycling processes, soil microbial activity, or plant nitrogen uptake mechanisms [40]. Furthermore, the significant negative correlation between *δ*^15^N isotope composition and humidity (*p* < 0.05) indicates that regions with higher humidity tend to have lower *δ*^15^N values. This could be due to the fact that *δ*^15^N isotope ratios are often influenced by soil moisture, which impacts nitrogen transformation processes like nitrification and denitrification. High moisture can increase nitrogen availability but can also lead to greater nitrogen losses through leaching, denitrification, or volatilization, potentially explaining the observed decrease in the *δ*^15^N values [41].

The *δ*^18^O and *δ*^2^H isotope compositions show a significant positive correlation with latitude (*p* < 0.05), which aligns with previous studies [28,42]. This is likely due to changes in temperature and precipitation along the latitudinal lines. As latitude increases, *δ*^18^O and *δ*^2^H isotopes become more enriched due to isotopic fractionation caused by temperature changes. Lower temperatures at higher latitudes often result in heavier isotopic compositions owing to reduced evaporation and precipitation recycling losses. *δ*^18^O and *δ*^2^H isotopes also show a significant negative correlation with temperature, precipitation, and humidity (*p* < 0.05). These findings are consistent with other studies [43] that have shown that *δ*^2^H and *δ*^18^O isotopes are influenced by latitude and climatic factors such as temperature, precipitation, and humidity. Lower temperatures deplete light isotopes in precipitation, while increases in precipitation and humidity alter isotope ratios by intensifying precipitation and moisture recycling effects. These climatic effects on isotope composition are critical to understanding the water cycle processes and ecosystem interactions across geographic regions [29]. The results of this study confirm earlier findings, such as those by Rozanski et al. [42], who demonstrated that *δ*^2^H and *δ*^18^O isotopes are significantly influenced by latitude and climatic factors such as temperature, precipitation, and humidity.

In conclusion, the stable isotope content in *R. tanguticum* is influenced by growing conditions and climatic factors. Environmental elements like longitude, latitude, precipitation, and humidity have the most significant impact on the isotope composition in rhubarb, subsequently affecting its chemical components [44]. Based on the above discussions, it can be inferred that regional climatic differences are the primary environmental factors shaping isotope compositions, which, in turn, lead to variations in the quality of *R. tanguticum* across different regions [35].

### 3.3. Geographical Origin Verification Based on Chemometric Models

For rhubarb traceability, either stable isotope values or elemental content can be utilized as variables in a discriminant model. However, combining stable isotope analysis with elemental analysis can enhance the accuracy and reliability of origin identification [18]. In this study, three classification models—linear discriminant analysis (LDA), k-nearest neighbor (k-NN), and partial least squares-discriminant analysis (PLS-DA)—were developed to determine the geographical origin of the *R. tanguticum* samples based on stable isotope values (*δ*^13^C, *δ*^15^N, *δ*^2^H, and *δ*^18^O) and elemental content (%C and %N) from the Gansu, Sichuan, and Qinghai regions. LDA is widely used for data classification due to its simplicity and effectiveness in identifying patterns. PLS-DA models are commonly employed to classify samples into predefined categories and predict the classification of unknown samples [45,46]. The k-NN algorithm, recognized for being lightweight, simple, and cost-effective, is particularly effective for small datasets and multi-class problems [47]. To develop the most robust model, the discriminative capabilities of all three methods were compared.

**Figure 4:**
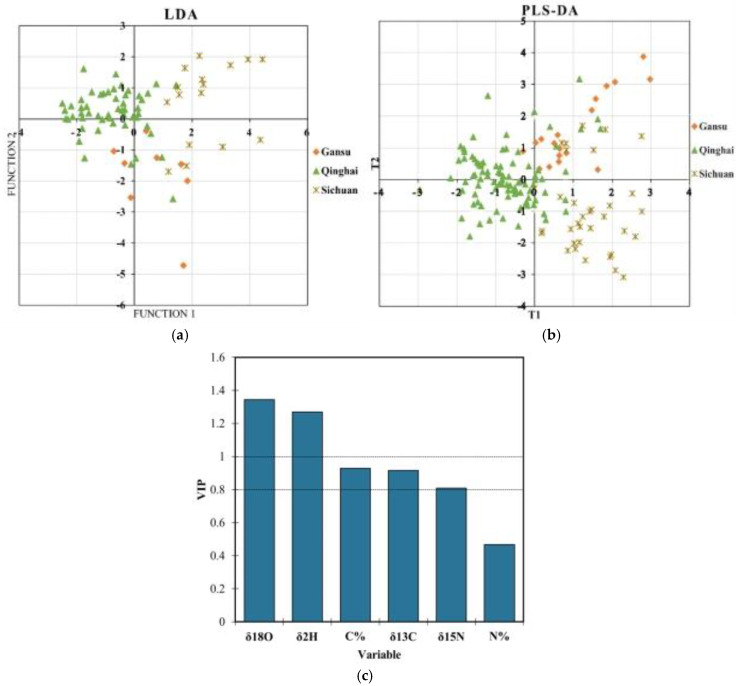
(**a**) Plot of the first two discriminant functions obtained with LDA. (**b**) PLS-DA modeling results based on stable isotopes for the geographical origin of *R. tanguticum* in different regions. (**c**) the variance importance plot (VIP).

Table 1 summarizes the results of the LDA, k-NN, and PLS-DA classifications of the rhubarb from the three regions. A total of 190 samples were used in the classification models, which were randomly divided into a training set of 145 samples and a validation set of 45 samples. A total of 16 samples from Gansu, 28 from Sichuan, and 101 from Qinghai were included in the LDA training set, while the validation set comprised 4 samples from Gansu, 17 from Sichuan, and 24 from Qinghai. The LDA model successfully classified the samples from the three regions, achieving 89.7% accuracy in the training set and 82.2% accuracy in the validation set. Gansu had the highest classification accuracy (93.8%) in the training set, while Qinghai had the highest accuracy (91.7%) in the validation set. Sichuan had the lowest classification accuracy (70.6%) in the validation set. The classification accuracy for the different regions was further confirmed by plotting the first two discriminant functions obtained from the LDA (Figure 4a).

In the k-NN model, the training set (n = 145) included 17 samples from Gansu, 35 from Sichuan, and 93 from Qinghai, while the validation set (n = 45) comprised 3 samples from Gansu, 10 from Sichuan, and 32 from Qinghai. The best results were obtained with k = 3. The k-NN model had an overall accuracy of 91.7% in the training set and 86.7% in the validation set for *R. tanguticum*. Qinghai had the best classification accuracy, with 96.8% in the training set and 93.8% in the validation set, followed by Gansu with 85.7% accuracy in the training set and 60.0% in the validation set. Interestingly, Gansu achieved 100% accuracy in the validation set despite a lower training accuracy of 76.5%.

For PLS-DA modeling, the raw data were randomly divided into a training set (n = 145) and a validation set (n = 45). The training set included 18 samples from Gansu, 34 from Sichuan, and 93 from Qinghai, while the validation set consisted of 2 samples from Gansu, 11 from Sichuan, and 32 from Qinghai. The PLS-DA plot (Figure 4b) showed that the samples from the three regions were approximately classified with good accuracy. The recognition results indicated that 82.1% of the original grouped samples were correctly classified in the training set, with the validation set yielding an accuracy of 91.1%. For Qinghai, all 45 samples were accurately classified, achieving 100% accuracy in the validation set. In contrast, Gansu had the lowest prediction accuracy, with 33.3% in the training set and 50% in the validation set. These findings align with previous studies by Zhao S. et al. [48], where the samples from Qinghai and Sichuan were classified with 100% accuracy, while Gansu samples showed lower accuracy (50%). The lower prediction accuracy for Gansu may be due to the heterogeneity of the data, potentially caused by environmental, genetic, or chemical variability in the region. Additionally, the small sample size from Gansu may not have provided enough data for the model to learn the region’s specific characteristics. Another possible reason is the insufficient representation of key features in the model, as PLS-DA is sensitive to the choice of variables. If the chosen attributes do not adequately capture biological or ecological variability in Gansu, the model may not have sufficient information to make accurate predictions.

Additionally, the variance importance plot (VIP) (Figure 4c) indicates that variables with a VIP score greater than one have a significant impact on the model and can serve as key discriminant markers. In this study, the isotopes *δ*^18^O and *δ*^2^H had VIP > 1, indicating their significant contribution to the accuracy of the geographical origin recognition for the rhubarb. Chemometric methods are essential for tracing origin, as different methods can yield varying results from the same data [18].

In this study, the results showed that the LDA, k-NN, and PLS-DA recognition models, based on the stable isotope values and elemental contents, were effective in accurately determining the geographical origin of *R. tanguticum*. The classification accuracy varied across the three regions, with Qinghai showing the highest accuracy, followed by Sichuan and Gansu. Among the three chemometric methods, k-NN delivered the highest accuracy, followed by PLS-DA, while LDA demonstrated the lowest accuracy.

## 4. Conclusions

This study demonstrated that the elemental content (%C and %N) and stable isotopes (*δ*^13^C, *δ*^1^^5^N, *δ*^2^H, and *δ*^1^^8^O) in *R. tanguticum* are significantly influenced by climatic factors across different production regions. Correlation analysis revealed that longitude, latitude, temperature, precipitation, and humidity had a notable effect on stable isotope compositions, emphasizing the impact of environmental factors on rhubarb’s isotopic components. Based on the stable isotope values and elemental content, LDA, k-NN, and PLS-DA models were developed for the three *R. tanguticum* growing regions, with overall classification accuracies ranging from 82.1% to 91.7%. The samples from the Qinghai region exhibited the highest classification accuracy and were clearly distinguished from those in Sichuan and Gansu across all three models. Additionally, the *δ*^1^^8^O and *δ*^2^H isotopes played a crucial role in enhancing the accuracy of the geographical origin determination. Therefore, the combination of stable isotope data with LDA, k-NN, and PLS-DA models offers a robust and efficient approach for accurately identifying the origin of rhubarb and can be applied to trace *R. tanguticum* from different regions. In the future, this method could assist in the traceability of other cultivars using these models.

## Figures and Tables

**Table 1:** Classification results of the LDA, k-NN, and PLS-DA of the rhubarb geographical origins by using the training and validation set in the different regions.

LDA	Regions	Predicted			Total
		Gansu	Sichuan	Qinghai	
Training	Gansu	15	0	1	16
	Sichuan	3	25	0	28
	Qinghai	9	2	90	101
	%, correct	93.8	89.3	89.1	89.7
Validation	Gansu	3	0	1	4
	Sichuan	2	12	3	17
	Qinghai	2	0	22	24
	%, correct	75.0	70.6	91.7	82.2
k-NN					
Training	Gansu	13	0	4	17
	Sichuan	1	30	4	35
	Qinghai	1	2	90	93
	%, correct	76.5	85.7	96.8	91.7
Validation	Gansu	3	0	0	3
	Sichuan	0	6	4	10
	Qinghai	0	2	30	32
	%, correct	100.0	60.0	93.8	86.7
PLS-DA					
Training	Gansu	6	1	11	18
	Sichuan	2	26	6	34
	Qinghai	4	2	87	93
	%, correct	33.3	76.5	93.6	82.1
Validation	Gansu	1	0	1	2
	Sichuan	1	8	2	11
	Qinghai	0	0	32	32
	%, correct	50.0	72.7	100.0	91.1

## Data Availability

The original contributions presented in the study are included in the article/Supplementary Material, further inquiries can be directed to the corresponding author.

## Supplementary Materials

The following supporting information can be downloaded at https://www.mdpi.com/article/10.3390/foods13193176/s1, Table S1: Characteristics of *R. tanguticum* sampling sites in Qinghai, Gansu, and Sichuan regions; Table S2: Climatic characteristics of *R. tanguticum* in three production regions.
