# Supplementary material for: Chemometric Discrimination of the Geographical Origin of Rheum tanguticum by Stable Isotope Analysis
Source: Foods. 2024 Oct 6;13(19):3176. doi: 10.3390/foods13193176 (PMC11475526; doi:10.3390/foods13193176)
Supplement: Supplementary file 1 [file foods-13-03176-s001.zip › foods-3202628-supplementary.pdf]

Table S1. Characteristics of *R. tanguticum* sampling sites in Qinghai, Gansu, and Sichuan regions (Zhao et al., 2023)

Province	Sites	Longitude, °E	Latitude, °N	Altitude, m
Gansu	ZhuoMin	103°30'02"E	34°30'46"N	3173
	MaQv	101°41'09"E	33°59'43"N	3634
	LuQv	102°28'13"E	34°38'36"N	3231
	ZhouQv	104°26'13"E	33°47'18"N	3106
Sichuan	JiuZhaiGou	103°42'29"E	33°07'38"N	3323
	SongPan	103°42'20"E	32°44'27"N	3798
	HongYuan	102°36'43"E	31°01'21"N	3319
	XiaoJin	102°19'43"E	31°39'23"N	3602
	SeDa	100°25'16"E	32°10'11"N	3796
	BaiYu	98°58'24"E	30°57'51"N	4282
	DeGe	99°00'43"E	31°53'31"N	4143
	ShiQv	98°20'12"E	32°59'36"N	3921
Qinghai	LeDu	102°09'23"E	36°19'58"N	3004
	JunGong	100°37'41"E	34°39'25"N	3326
	JiKa	100°15'39"E	32°49'35"N	3869
	Baiyu	100°46'45"E	33°14'14"N	4016
	DaRi	99°28'58"E	33°42'04"N	4180
	GanDe	100°15'34"E	33°39'07"N	3865
	TongDe	100°48'52"E	34°47'05"N	3381
	ZeKu	101°49'02"E	35°04'25"N	3545
	TongRen	101°39'42"E	34°32'32"N	3679
	HuaLong	101°59'06"E	35°22'59"N	3035
	MaQin	100°27'28"E	34°32'03"N	3685
	MaQin	100°24'28"E	34°28'39"N	4167
	MaQin	100°24'27"E	34°28'38"N	3997

MaQin	100°29'18.7"E	34°21'42.7"N	3948
GanDe	100°29'18.8"E	34°21'43"N	3828
JiuZhi	101°01'47"E	33°47'43"N	3610
JiuZhi	101°28'37"E	33°25'39"N	3532
BanMa	100°39'10"E	32°40'29"N	3992
BanMa	100°55'09"E	32°44'39"N	3463
BanMa	100°45'57"E	32°55'17"N	3437
DaRi	100°35'46"E	33°5'42.36"N	3644
DaRi	100°25'02"E	33°16'40"N	4157
XingHai	99°59'47"E	35°53'30"N	3246
GongHe	100°16'32"E	36°14'16"N	2861
NangQian	95°48'30"E	32°22'16"N	4400

Table S2. Climatic characteristics of *R. tanguticum* in three production regions (August- September, 2018)

Parameters	Region					
	Gansu		Sichuan		Qinghai	
	August 2018	September 2018	August 2018	September 2018	August 2018	September 2018
Average temperature, °C	22.77°C	16.7°C	26.87°C	20.43°C	20.77°C	15.87°C
Average precipitation, mm	1.02mm	0.25mm	2.05mm	3.34mm	0.25mm	0.13mm
Average humidity, %	58%	53%	73%	85%	34%	26%

https://weatherandclimate.com/
